# Erweiterte Hersteller- und Produktverantwortung im Abwasserrecht – Rechtliche Eckpunkte für eine verursachergerechte Lastentragung bei der Vermeidung und Reduzierung von Spurenstoffen im Gewässer

**DOI:** 10.1007/s10357-020-3784-0

**Published:** 2021-01-19

**Authors:** Michael Reinhardt

**Affiliations:** Lehrstuhl für Öffentliches Recht, Universität Trier, Trier, Deutschland

## Abstract

Der Beitrag befasst sich mit der im geltenden Recht bislang allenfalls randständig aufgegriffenen Problematik der zunehmenden Belastung der Gewässer mit sog. Spurenstoffen. Er diskutiert einen Regelungsansatz de lege ferenda, der sich an der abfallrechtlichen Konstruktion der Hersteller- und Produktverantwortung orientiert.

### 1. Einführung

Die Einträge sog. Spurenstoffe (z.B. Rückstände von Kunststoffprodukten, Arzneimitteln, Pflanzenschutzmitteln, Bioziden) in die Gewässer zählen zu den bislang rechtlich nicht abschließend geklärten Fragen des Gewässerschutzrechts. Rechtsverbindliche Emissions- oder Immissionswerte, auf Grund derer ordnungsrechtliche Maßnahmen getroffen werden können, sind derzeit weder im europäischen noch im deutschen Recht flächendeckend angeordnet. Im Rahmen der sog. Spurenstoffstrategie des Bundes, die darauf abzielt, den Eintrag von relevanten Spurenstoffen in die aquatische Umwelt zu vermeiden oder zu reduzieren, werden seit geraumer Zeit intensiv und kontrovers vor allem alternative Instrumente der Verhaltenssteuerung erörtert, unter denen beispielhaft eine entsprechende Anpassung des Abwasserabgabenrechts oder die Finanzierung zusätzlicher Maßnahmen der Abwasserbehandlung über einen verursachergetragenen Fonds zu nennen sind. Darüber hinaus hat die Konferenz der Umweltminister (UMK) im Mai 2019 das Bundesministerium für Umwelt, Naturschutz und nukleare Sicherheit (BMU) gebeten, bei der weiteren Konkretisierung der Strategie insbesondere Aspekte der Produkt- und Herstellerverantwortung stärker in den Blick zu nehmen. Der nachstehende Beitrag befasst sich mit den rechtlichen Rahmenbedingungen für die Einführung einer am Vorbild der unionsrechtlich vorgegebenen erweiterten Herstellerverantwortung im Abfallrecht orientierten Regelung auch für das Wasserrecht, um einen Beitrag zur Entlastung der kommunalen Abwasserbeseitigung zu leisten.

### 2. Rechtliche Ausgangslage

#### *2.1 Einfach-gesetzliches Bundesrecht*

##### 2.1.1 Wasserhaushaltsgesetz

Die rechtliche Grundlage für den Schutz der Gewässer vor anthropogenen Einträgen bildet zunächst das Wasserhaushaltsgesetz mit seinen Regelungen über die öffentlich-rechtliche Bewirtschaftung. Nach § 8 Abs. 1 WHG bedarf jede Benutzung eines Gewässers im Sinne des § 9 WHG einer wasserbehördlichen Erlaubnis oder Bewilligung. Für die hier in Rede stehende Abwasserbeseitigung ist in erster Linie auf § 9 Abs. 1 Nr. 4 WHG abzustellen, der das Einbringen und Einleiten von Stoffen als zulassungspflichtige Benutzung definiert. § 12 Abs. 1 WHG regelt sodann zwingende gesetzliche Versagungsgründe, die vor allem auf den Schutz der Gewässer vor schädlichen Gewässerveränderungen im Sinne des § 3 Nr. 10 WHG und die Erfüllung der einschlägigen öffentlich-rechtlichen Vorschriften drängt. Unionsrechtlich vorgeschrieben ist zudem allgemein die Erreichung eines guten Zustands resp. eines guten Potenzials, wie er im Einzelnen in den §§ 27, 28, 44, 47 WHG gewässertypspezifisch umschrieben ist. Im Übrigen steht die Zulassung der Benutzung nach § 12 Abs. 2 WHG im Ermessen der zuständigen Wasserbehörde.

Insbesondere im Recht der Abwasserbeseitigung verfolgt das Wasserhaushaltsgesetz einen sog. kombinierten Ansatz[1], der neben der Gewährleistung bestimmter Qualitätsanforderungen im Gewässer (Immissionsprinzip) auch konkrete Vorgaben an die Beschaffenheit des eingeleiteten Abwassers formuliert (Emissionsprinzip). Diese werden für Direkteinleitungen in ein Gewässer durch die Voraussetzungen des § 57 WHG auf dem allgemeinen Niveau des Stands der Technik postuliert, während § 58 WHG die Einhaltung dieser Vorgaben auch für Indirekteinleitungen in öffentliche Abwasseranlagen sicherstellt.[2] Für die Einleitung in private Abwasseranlagen vervollständigt § 59 WHG die Regelungslage.

Nicht hingegen definiert das Wasserhaushaltsgesetz die vollzugsfähige Konkretisierung der so allgemein vorgegebenen Immissions- und Emissionsziele für bestimmte Stoffe und Stoffgruppen selbst, sondern überlässt dies dem nachgeordneten Verordnungsrecht, dem zugleich auch die Aufgabe der Transformation einschlägiger Vorgaben, insbesondere die Übernahme numerischer Grenzwerte des öffentlichen Gewässerschutzrechts der Europäischen Union, zufällt.[3]

Soweit diese verordnungsrechtlichen Konkretisierungen der wasserhaushaltsgesetzlichen Schutz- und Vorsorgestandards[4] bei einer Abwassereinleitung eingehalten werden, besteht gemäß § 12 Abs. 2 WHG grundsätzlich kein Anspruch auf Erteilung einer Erlaubnis, sondern lediglich auf ermessensfehlerfreie Entscheidung.[5] Daraus folgt, dass die zuständige Wasserbehörde berechtigt sein kann, strengere Anforderungen per Bescheid festzulegen, wenn und

Prof. Dr. Michael Reinhardt, LL.M. (Cantab.),
Lehrstuhl für Öffentliches Recht an der Universität Trier
und Direktor des Instituts für Deutsches und
Europäisches Wasserwirtschaftsrecht,
Universität Trier,
Trier, Deutschland

1) Dazu z.B. *Breuer/Gärditz*, Öffentliches und privates Wasserrecht, 4. Aufl. 2017, Rdnr. 182, 700 ff.; *Durner*, Wasserrecht, in: Rehbinder/Schink (Hrsg.), Grundzüge des Umweltrechts, 5. Aufl. 2018, Kap. 9 Rdnr. 68 ff.
2) Siehe z.B. *Czychowski/Reinhardt*, Wasserhaushaltsgesetz, Kommentar, 12. Aufl. 2019, § 58 Rdnr. 3; *Nisipeanu*, in: Berendes/Frenz/Müggenborg (Hrsg.), Wasserhaushaltsgesetz, Kommentar, 2. Aufl. 2017, § 58 Rdnr. 11 ff., 34; jeweils m. w. N.
3) Dazu sogleich 2.2.
4) Zu dieser Differenzierung statt anderer *Salzwedel*, in: Isensee/Kirchhof (Hrsg.), Handbuch des Staatsrechts der Bundesrepublik Deutschland, Bd. IV, 3. Aufl. 2006, § 97 Rdnr. 11 ff.
5) BVerfG, Beschl. v. 15.7.1981 – 1 BvL 77/78, BVerfGE 58, 300, 347; *Czychowski/Reinhardt*, WHG, § 12 Rdnr. 33 m. w. N.

soweit dies im einzelnen Fall gerechtfertigt ist.[6] Hiervon gedeckt werden soll nach verbreiteter Ansicht auch das Ziel der Wasserbehörde, „anspruchsvollere ökologische Bewirtschaftungsziele zu erreichen, insbesondere die Besiedlung des Gewässers mit pflanzlichem und tierischem Leben zu verbessern".[7] Berechtigt das wasserbehördliche Bewirtschaftungsermessen dazu, auch bei Fehlen der absoluten Versagungsgründe des § 12 Abs. 1 WHG die Erteilung der Zulassung vollständig zu versagen, so ist es nach dem verhältnismäßigkeitsrechtlichen Gedanken des milderen Mittels erst recht zulässig, die Gewässerbenutzung zu gestatten, dabei aber strengere, d. h. über die gesetzlichen Anforderungen hinausgehende Vorgaben zu formulieren, etwa weil die Besonderheiten eines atypischen Falls[8] dies im betroffenen Wasserkörper gebieten. Dabei bleiben aber in rechtssystematischer Hinsicht die bundesrechtlich definierten Standards als solche dem Zugriff der Landesexekutive schon aus kompetenzrechtlichen Gründen verschlossen.[9] Die Wasserwirtschaftsverwaltung kann daher nicht die in Wasserhaushaltsgesetz und nachgeordnetem Verordnungsrecht formulierten Anforderungsniveaus des „guten Zustands" und des „Stands der Technik" selbst neu und strenger definieren, indem sie auf dem Wege etwaig auch erlassgelenkter Bescheidpraxis eine eigenständige ordnungsrechtliche Spurenstoffstrategie verfolgt; lediglich ist sie im Rahmen des Bewirtschaftungsermessens berechtigt, zumindest partiell einen „sehr guten Zustand" eines Wasserkörpers anzustreben oder strengere Emissionswerte als nach dem Stand der Technik vorgeschrieben anzuordnen, wenn und soweit dies im Einzelfall einer Abwassereinleitung in einen bestimmten Wasserkörper tatsächlich zureichend begründet werden kann. Zu bedenken ist auch, dass das wasserhaushaltsgesetzliche Bewirtschaftungsermessen nach Herkommen, Systematik, Zielsetzung und verfassungsrechtlicher Rechtfertigung als nutzungsbezogenes Verteilungsermessen, nicht aber als ökologisches Schutzverstärkungsermessen ausgestaltet ist, so dass eine auf ausschließlich gewässerökologische Beweggründe gestützte Ermessensentscheidung zur Spurenstoffelimination von der Ermächtigung des § 12 Abs. 2 WHG nicht mehr gedeckt wird.[10]

##### 2.1.2 Abwasserabgabengesetz

Als flankierende ökonomische Steuerung der Gewässerbelastung durch Abwassereinleitungen fungiert sodann das Abwasserabgabengesetz des Bundes, das durch eine verursachungsbezogene Lenkung des Einleiteverhaltens zu Reduzierung und Vermeidung der Schadstoffeinträge beitragen will.[11] Dabei wird zur Ermittlung der Abgabenlast und damit zur Verhaltenssteuerung des Abgabenpflichtigen nach § 4 AbwAG grundsätzlich an die im wasserbehördlichen Bescheid jeweils zugelassenen Schadstofffrachten angeknüpft. Eine gegenwärtig bisweilen diskutierte und rechtlich durchaus denkbare spezifische Regelung zur abgabenrechtlichen Steuerung des Spurenstoffeintrags in die Gewässer fehlt in dem Gesetz derzeit indes.

#### *2.2 Konkretisierendes Verordnungsrecht*

Im Hinblick auf die bereits angesprochenen untergesetzlichen Konkretisierungen der abstrakten Immissions- und Emissionsstandards des Wasserhaushaltsgesetzes ist in dem hier interessierenden Zusammenhang insbesondere auf die spezifischen verordnungsrechtlichen Vorgaben für die Qualität der Oberflächengewässer[12] und des Grundwassers[13] sowie auf die Mindestanforderungen an das Einleiten von Abwasser in Gewässer[14] hinzuweisen. Der zwar umfängliche und zudem im Zusammenwirken mit der Normgebung der Europäischen Union sukzessive angepasste und erweiterte Bestand rechtlich reglementierter Stoffe und Stoffgruppen zur Immissions- und Emissionssteuerung spiegelt jedoch die Gesamtheit der heute naturwissenschaftlich nachweisbaren und als gesundheitlich oder ökologisch problematisch bewerteten anthropogenen Einträge in die Gewässer nur unvollständig wider. Insbesondere ermöglichen die in jüngerer Zeit erheblich fortgeschrittenen Mess- und Analyseverfahren neue Erkenntnisse über den tatsächlichen qualitativen Gewässerzustand, der über die Kenntnisnahme und Bewertung der verordnungsrechtlich explizit aufgegriffenen stofflichen Einträge deutlich hinausgeht. Dies gilt vor allem für die hier im Focus stehenden verschiedenartigen Spurenstoffe, deren rechtliche Bewältigung derzeit Gegenstand zahlreicher, noch nicht abgeschlossener Konsultationen auf europäischer und nationaler Ebene ist.

#### *2.3 Bewertung: Grenzen des Ordnungsrechts*

Bereits dieser kursorische Blick auf die wasserrechtlichen Grundlagen des immissions- und emissionsbezogenen Gewässerschutzes hat die Grenzen des Ordnungsrechts als Instrument der angemessenen Bewältigung der Spurenstoffproblematik aufgezeigt:

Zum einen mögen zwar sukzessive erweiterte spezifische normative Standards dem Spurenstoffeintrag grundsätzlich entgegenzuwirken in der Lage sein, doch erweist sich die Reglementierung auf dem Verordnungsweg als vielfach zu schwerfällig, um mit dem raschen naturwissenschaftlichen Erkenntnisfortschritt und dem daraus hergeleiteten Handlungsbedarf mitzuhalten.[15] Dies gilt umso mehr, als die rechtlich verbindliche Anordnung eines konkreten Grenz- oder Schwellenwerts regelmäßig individuelle Handlungsfreiheiten beschränkt und damit grundrechtseingreifende Wirkung aufweist, so dass sie aus rechtsstaatlichen Gründen der zureichenden, insbesondere dem Verhältnismäßigkeitsgrundsatz genügenden Rechtfertigung bedarf.[16] Da indes Gesundheitsgefährlichkeit und Umweltrelevanz zahlreicher Stoffe und Stoffgruppen gerade bei quantitativ gering-

6) Siehe OVG Münster, Beschl. v. 30. 9. 2015 – 20 A 2660/12, ZfW 2016, 175, 177 ff.; OVG Lüneburg, Urt. v. 20. 11. 2014 – 13 LC 140/13, NVwZ-RR 2015, 413, 414 f.; ebenso *Breuer/Gärditz*, Öffentliches und privates Wasserrecht, Rdnr. 615; *Czychowski/Reinhardt*, WHG, § 57 Rdnr. 14; *Durner*, RdWEWi 42, 2014, 13 ff.; *Elgeti*, W+B 2015, 137, 140; *Meyer*, W+B 2016, 86, 88 ff.

7) So *Durner*, in: Rehbinder/Schink (Hrsg.), Grundzüge des Umweltrechts, Kap. 9 Rdnr. 39; ebenso OVG Lüneburg, Urt. v. 20. 11. 2014 – 13 LC 140/13, NVwZ-RR 2015, 413, 414 unter Hinweis u. a. auf den Natur- und Landschaftsschutz; *Elgeti*, W+B 2015, 137, 140.

8) VGH Mannheim, Urt. v. 8. 11. 2011 – 3 S 1728/09, BeckRS 2012, 45894; ähnlich schon VGH Kassel, Urt. v. 26. 2. 2003 – 5 UE 2304/01, BeckRS 2003, 22775, zum Abwasserabgabenrecht.

9) In diesem Sinn auch *Durner*, RdWEWi 42, 2014, 20 ff.

10) Dazu näher *Reinhardt*, NVwZ 2017, 1000 ff.

11) Siehe etwa *Breuer/Gärditz*, Öffentliches und privates Wasserrecht, Rdnr. 83 ff.; *Rehbinder*, Ziele, Grundsätze, Strategien und Instrumente, in: Rehbinder/Schink (Hrsg.), Grundzüge des Umweltrechts, Kap. 3 Rdnr. 284 ff.

12) Verordnung zum Schutz der Oberflächengewässer (Oberflächengewässerverordnung – OGewV) v. 20. 6. 2016, BGBl. I S. 1373.

13) Verordnung zum Schutz des Grundwassers (Grundwasserverordnung – GrwV) v. 9. 11. 2010, BGBl. I S. 1513.

14) Verordnung über Anforderungen an das Einleiten von Abwasser in Gewässer (Abwasserverordnung – AbwV) in der Fassung der Bekanntmachung v. 17. 6. 2004, BGBl. I S. 2625.

15) Insbesondere ist in diesem Zusammenhang auf den verfahrensrechtlich komplexen Sevilla-Prozess nach Art. 75 der Industrieemissionenrichtlinie 2010/75/EU zur Verabschiedung der BVT-Merkblätter und BVT-Schlussfolgerungen im Abwasserrecht hinzuweisen; siehe dazu z. B. *Czychowski/Reinhardt*, WHG, § 54 Rdnr. 36 ff.; *Nisipeanu*, in: Berendes/Frenz/Müggenborg (Hrsg.), WHG, § 54 Rdnr. 45 ff.

16) Siehe z. B. *Durner*, W+B 2014, 115, 119 f.; *Reinhardt*, NuR 2018, 289, 296; siehe auch *Depenheuer*, Politik durch Zahlen, und *Posser*, Verwaltungsgerichtliche Kontrolle der behördlichen Grenzwertsetzung, jeweils in: Reinhardt (Hrsg.), Der Grenzwert im Wasserrecht, Trierer Wasserwirtschaftsrechtstag 2019, S. 21 ff., 53 ff.

fügigen Einträgen heute noch nicht abschließend bewertet werden können, unterliegt ein ordnungsrechtlicher Ansatz in besonderer Weise den verfassungsrechtlich gebotenen Einschränkungen für vorsorgende Regelungen.[17]

Zum anderen ist auch das wasserbehördliche Ermessen nach § 12 Abs. 2 WHG schon strukturell nicht geeignet, einen flächendeckenden Schutz der Gewässer vor dem Eintrag von Spurenstoffen zu bewirken. Nichts anderes gilt im Übrigen für die übergeordnete Ermessenslenkung auf dem Weg der wasserwirtschaftlichen Planung nach den §§ 82 ff. WHG[18], deren Instrumentarium mangels Rechtsnormcharakter zu eingreifenden Maßnahmen generell-abstrakter Art nicht ermächtigt.[19]

Nur konsequent erstrecken sich daher die gegenwärtigen Diskussionen über die Spurenstoffproblematik auch auf alternative rechtliche Bewältigungsstrategien, die neben anderem auf die nachfolgend zu erörternde Implementierung einer erweiterten Herstellerverantwortung im Abwasserrecht zielt.[20] Ziel kann es hierbei freilich allein sein, eine die skizzierten strukturellen Defizite des klassischen Ordnungsrechts vermeidende rechtliche Handhabung zu finden, nicht hingegen wäre auf diesem Weg eine Derogation der rechtsstaatlichen und grundrechtlichen Anforderungen an die Beschränkung individueller Freiheiten der Normadressaten zulässig.

### 3. Modell: Erweiterte Hersteller- und Produktverantwortung im Abfallrecht

#### *3.1 Europarechtliche Vorgaben*

Als unionsrechtliche Grundlage der erweiterten Hersteller- und Produktverantwortung dienen zunächst die – im Übrigen dem deutschen Vorbild nachempfundenen[21] – Regelungen der Abfallrahmenrichtlinie 2008/98/EG[22]. In Konkretisierung des in Art. 191 Abs. 2 UAbs. 1 S. 2 AEUV enthaltenen Verursacherprinzips[23] werden die Mitgliedstaaten zur Formulierung von Herstellerpflichten angehalten, die nach Art. 8 Abs. 2 UAbs. 1 AbfRRL neben anderen Maßnahmen umfassen, die darauf hinwirken, dass bei Herstellung von Produkten nachteilige Umweltfolgen und die Entstehung von Abfällen verringert werden. Gemäß Art. 8 Abs. 3 AbfRRL sind dabei die technische und wirtschaftliche Durchführbarkeit und die Gesamtauswirkungen auf die Umwelt und die menschliche Gesundheit, die sozialen Folgen und das ordnungsgemäße Funktionieren des europäischen Binnenmarkts zu berücksichtigen. Der später ergänzte Art. 8a AbfRRL[24] beschreibt sodann spezifische Mindestanforderungen an Regime der erweiterten Herstellerverantwortung, zu denen auch die Auferlegung finanzieller Lasten zur Kostendeckung u. a. bestimmter Abfallbehandlungsmaßnahmen zählt.

Für den Sonderfall der Bekämpfung der Umweltbelastung durch Kunststoffe knüpft die neue Kunststoffproduktrichtlinie 2019/904/EU[25] an dieser erweiterten Herstellerverantwortung an, indem nach Art. 8 Abs. 2, 3 der Richtlinie die Hersteller von im einzelnen enumerierten Kunststoffartikeln in differenzierter Weise zur Tragung u. a. der Kosten für Sammlung, Beförderung und Behandlung der daraus entstehenden Abfälle verpflichtet werden sollen.[26] Erklärtes Ziel der Regelung ist die Deckung der Kosten von Abfallvermeidung und Abfallbewirtschaftung, einschließlich der Säuberung der Umwelt von Plastikmüll[27], erneut als bereichsspezifisch konkretisierende Operationalisierung des abstrakten primärrechtlichen Verursacherprinzips.

#### *3.2 Deutsches Transformationsrecht*

Den Kern der im deutschen Recht als Produktverantwortung bezeichneten Umsetzung bilden die §§ 23 ff. KrWG. Sie setzen in Konkretisierung verschiedener umweltrechtlicher Prinzipien[28] bereits beim Herstellungsprozess von Produkten an und nehmen den Erzeuger als Verursacher in die Pflicht, zur Vermeidung sowie zur umweltverträglichen Verwertung und Beseitigung von Abfällen beizutragen.[29] Dem in § 23 Abs. 1 KrWG formulierten Grundgedanken folgen in § 23 Abs. 2 KrWG exemplarisch aufgezählte einzelne Bestandteile der Produktverantwortung, die vom Entwicklungs- und Herstellungsprozess bis zur Rücknahme mit nachfolgender umweltverträglicher Verwertung oder Beseitigung reichen. Die gesetzlichen Pflichten gehen dabei über einen rein appellativen Charakter hinaus, bedürfen aber zur effektiven Durchsetzung der Nachschärfung durch spezifisches Gesetzes- oder Verordnungsrecht.[30]

Im Gegensatz zu diesem umfassenden Normenbestand zur Umsetzung der erweiterten Herstellerverantwortung nach Art. 8 AbfRRL fehlen im deutschen Recht bislang naturgemäß transformationsrechtliche Regelungen für die Implementierung der unionsrechtlichen Herstellerverantwortung für bestimmte Kunststoffprodukte. Gemäß Art. 17 Abs. 1 UAbs. 1 der Kunststoffproduktrichtlinie sind die Mitgliedstaaten bis zum 3. Juli 2021 zum Erlass der erforderlichen Rechts- und Verwaltungsvorschriften verpflichtet, nach Art. 17 UAbs. 2 der Richtlinie sind die

17) Dazu schon *Salzwedel*, in: Isensee/Kirchhof (Hrsg.), HStR IV, § 97 Rdnr. 13; näher auch unten 4.2.2.
18) Dazu z. B. *Czychowski/Reinhardt*, WHG, § 12 Rdnr. 49; *Hasche*, ZfW 2004, 144 ff.; *Schmid*, in: Berendes/Frenz/Müggenborg (Hrsg.), WHG, § 12 Rdnr. 70 ff.
19) *Appel*, ZUR Sh 2002, 129, 136; *Breuer/Gärditz*, Öffentliches und privates Wasserrecht, Rdnr. 794; *Czychowski/Reinhardt*, WHG, § 82 Rdnr. 11 f., § 83 Rdnr. 8; *Durner*, W+B 2016, 115, 116; *Reinhardt*, NuR 2018, 289, 295.
20) So ausdrücklich z. B. der Beschluss der 92. Umweltministerkonferenz vom 10. 5. 2019 zu TOP 35 „Abschluss des Stakeholder-Dialogs zur Spurenstoffstrategie des Bundes", Endgültiges Ergebnisprotokoll vom 11. 6. 2019, S. 66; siehe auch schon das Ergebnispapier der Phase 2 des Stakeholder-Dialogs „Spurenstoffstrategie des Bundes" zur Umsetzung von Maßnahmen für die Reduktion von Spurenstoffeinträgen in die Gewässer vom März 2019, S. 20 ff.
21) *Mann*, in: Versteyl/Mann/Schomerus, Kreislaufwirtschaftsgesetz, Kommentar, 4. Aufl. 2019, § 23 Rdnr. 36; *Prelle*, in: Schmehl/Klement (Hrsg.), Gemeinschaftskommentar zum Kreislaufwirtschaftsgesetz, 2. Aufl. 2019, § 23 Rdnr. 6.
22) Richtlinie 2008/98/EG vom 19. 11. 2008 über Abfälle und zur Aufhebung bestimmter Richtlinien, ABl. 2008 L 312, S. 3 (AbfRRL).
23) *Konzak*, in BeckOK Umweltrecht, 55. Edition 2020, § 23 KrWG Rdnr. 1.1; *Petersen*, NVwZ 2009, 1063, 1071.
24) Eingefügt durch Richtlinie 2018/851/EU vom 30. 5. 2018 zur Änderung der Richtlinie 2008/98/EG über Abfälle, ABl. 2018 L 150, S. 109.
25) Richtlinie 2019/904/EU vom 5. 6. 2019 über die Verringerung der Auswirkungen bestimmter Kunststoffprodukte auf die Umwelt, ABl. 2019 L 155, S. 1; siehe dazu *Falke*, ZUR 2019, 562 f.
26) Der Ansatz wird förmlich unterstützt in der Stellungnahme des Europäischen Wirtschafts- und Sozialausschusses zum Kommissionsvorschlag vom 19. 2. 2019, ABl. 2019 C 62, S. 207, 211 (4.11); zur (überwiegend) positiven Reaktion auch der betroffenen Hersteller und sonstigen Interessenträger siehe die Entwurfsbegründung COM(2018) final, S. 11.
27) So die Entwurfsbegründung COM(2018) 340 final, S. 5, 14 ff.; siehe zudem die ausdrückliche Festschreibung des Kostendeckungsprinzips in Art. 8 Abs. 4 Richtlinie 2019/904/EU.
28) *Kloepfer*, Umweltrecht, 4. Aufl. 2016, § 21 Rdnr. 188: Verursacherprinzip, Cradle-to-grave-Prinzip, Nachsorgeprinzip; näher unten 4.1.
29) Siehe dazu neben den Kommentierungen der §§ 23 ff. KrWG z. B. *Kloepfer*, Umweltrecht, § 21 Rdnr. 187 ff.
30) *Konzak*, in: BeckOK Umweltrecht, § 23 KrWG Rdnr. 16; *Prelle*, in: Schmehl/Klement (Hrsg.), KrWG, § 23 Rdnr. 12; *Mann*, in: Versteyl/Mann/Schomerus, KrWG, § 23 Rdnr. 8; *Tünnesen-Harmes*, in: Jarass/Petersen (Hrsg.), Kreislaufwirtschaftsgesetz, Kommentar, 2014, § 23 Rdnr. 6.

Maßnahmen zur Erfüllung der erweiterten Herstellerverantwortung nach Art. 8 der Richtlinie grundsätzlich spätestens[31] ab dem 31. Dezember 2024 anzuwenden. Ein noch nicht abgestimmter Referentenentwurf zur Umsetzung des EU-Legislativpakets zur Kreislaufwirtschaft ist mit Stand vom 5. August 2019 auf dem Internetauftritt des Bundesministeriums für Umwelt, Naturschutz und nukleare Sicherheit veröffentlicht.

#### *3.3 Bewertung: Übertragbarkeit auf das Abwasserrecht*

Die Frage der grundsätzlichen Übertragbarkeit des hier nur zu skizzierenden Regelungsmodells der erweiterten Hersteller- und Produktverantwortung im Abfallrecht auf das Recht der Abwasserbeseitigung führt zunächst in das Verhältnis der beiden Rechtsmaterien zueinander, das Rechtsprechung und Schrifttum seit Jahrzehnten beschäftigt und auf absehbare Zeit auch weiter und ohne erkennbare Aussicht auf eine klare Grenzziehung beschäftigen wird.[32] Üblicherweise sind derartige, in der Rechtsordnung durchaus nicht seltene persistente Abgrenzungsschwierigkeiten ein Indiz für eine normativ artifizielle Unterscheidung zwischen unterschiedlichen, einander tatsächlich überlagernden oder tatsächlich ineinander übergehenden Regelungsgegenständen in der Absicht zumindest teilweise unterschiedlicher rechtlicher Bewältigung. Daraus folgt zunächst zwanglos, dass das Konstrukt der erweiterten Hersteller- und Produktverantwortung jedenfalls nicht in einer derart strikten systematischen oder tatsächlichen Ausschließlichkeit mit dem Kreislaufwirtschaftsrecht verbunden ist, dass sich die Überlegung einer Implementierung eines solchen Konstrukts auch im Abwasserrecht rechtsmethodisch *a priori* verbietet. Zudem erfasst schon die erweiterte Herstellerverantwortung nach der Kunststoffproduktrichtlinie 2019/904/EU explizit u. a. auch solche Produkte, die wie beispielsweise Feuchttücher[33] und Tabakprodukte[34] faktisch verbreitet in das Abwasser gelangen, so dass sich eine entsprechende Erstreckung des Rechtsregimes auf Bereiche jenseits des Abfallrechts im engeren Sinn durchaus aufdrängt.

Diese grundsätzliche systematische Übertragbarkeit belegt zudem die Überprüfung der bundesrechtlichen Grundsatzbestimmung des § 23 Abs. 1 KrWG. In ihr wird eine individuelle unternehmerische Verantwortung im Sinne eines allgemeinen kategorischen Imperativs[35] formuliert, die zu Vermeidung und Verminderung von Abfällen aus den von ihnen in Verkehr gebrachten Produkten sowie zur umweltverträglichen Verwertung und Beseitigung beitragen soll. Das Gesetz setzt dabei schon in den Stadien der Entwicklung, Herstellung und Be- resp. Verarbeitung an, um die Erfüllung der kreislaufwirtschaftsrechtlichen Ziele gewissermaßen an der Quelle[36] zu unterstützen, wie § 23 Abs. 1 S. 1 KrWG selbst explizit formuliert. So gesehen bestehen damit keine Bedenken, eine entsprechende Regelung *mutatis mutandis* auch für das Abwasserrecht zu schaffen, um gezielt an der Einleitung von Stoffen in ein Gewässer oder in eine öffentliche oder private Abwasseranlage anzusetzen.

Allerdings bleibt die praktische Steuerungskraft einer derartigen Grundsatzbestimmung wie gesehen äußerst beschränkt[37] und bedarf daher der Konkretisierung durch die Formulierung geeigneter Pflichten des Abwasserproduzenten. In diesem Sinn meint hier und im Folgenden der Begriff des Abwasserproduzenten das Unternehmen, das betriebliches Abwasser unmittelbar in ein Gewässer oder in eine Abwasseranlage einleitet oder das Produkte herstellt oder in Verkehr bringt, von denen Bestandteile oder Reststoffe in das Abwasser gelangen können resp. typischerweise gelangen; hiervon begrifflich zu trennen sind die Träger der Abwasserbeseitigungspflicht im Sinne des § 56 WHG in Verbindung mit dem jeweiligen Landesrecht. Orientiert man sich insoweit sodann wiederum am Modell des Kreislaufwirtschaftsgesetzes, so erweisen sich die in § 23 Abs. 2 KrWG einfach-gesetzlich begründeten Einzelpflichten als nurmehr teilweise auf die Regelung von Spurenstoffen im Abwasser übertragbar. So mögen etwa die Verpflichtungen zu einem spurenstoffarmen oder -freien Produktdesign (entsprechend § 23 Abs. 2 Nr. 1 KrWG) und zur Kennzeichnung spurenstoffhinterlassender Erzeugnisse (entsprechend § 23 Abs. 2 Nr. 3 KrWG) auch für das Abwasserrecht normierbar und durchsetzbar sein, doch gilt dies nicht oder allenfalls eingeschränkt beispielsweise für das Gebot des Vorrangs des Einsatzes von Sekundärrohstoffen, für Hinweise auf Verwertungsmöglichkeiten und Pfandregelungen sowie für Rücknahmepflichten (entsprechend § 23 Abs. 2 Nr. 2, 4, 5 KrWG). Nach einer möglichen Grundsatzentscheidung für die Implementierung einer erweiterten Herstellerverantwortung im Abwasserrecht wären dann mithin auf einer zweiten Stufe die betroffenen Prozesse und Produkte im Einzelnen zu identifizieren und auf ihr Gefährdungspotenzial im Abwasser sowie auf geeignete praktische Anknüpfungspunkte für die Formulierung konkreter Rechtspflichten hin zu überprüfen. Dies bedarf indes einer differenzierten branchen-, produkt- und stoffspezifischen technischen Bewertung, die im Rahmen einer rein juristischen Untersuchung nicht geleistet werden kann. Nur in einem solchen mehrstufigen, interdisziplinären Vorgehen könnte eine den tatsächlichen Besonderheiten spurenstoffhaltigen Abwassers Rechnung tragende eigenständige und auch verfassungsrechtlich tragfähige Regelung der Herstellerpflichten entwickelt und normativ fixiert werden.

Das Beispiel des Kreislaufwirtschaftsrechts lehrt wie gesehen insoweit aber auch, dass selbst derartige gesetzlichen Konkretisierungen noch keine hinreichend vollzugstauglichen Maßstäbe und Verhaltenspflichten bereitstellen, so dass zusätzliche, spezifisch diversifizierte Detailregelungen ergänzt werden müssen[38], die in dem hier interessierenden Zusammenhang mindestens für die Bereiche Mikroplastik, Arzneimittelrückstände und Spurenstoffe von Pflanzenschutzmitteln und Bioziden im Abwasser zu schaffen sein werden.

Im geltenden Kreislaufwirtschaftsrecht bislang nur randständig aufgegriffen ist dagegen die Frage nach aus einer gesetzlichen Produktverantwortung etwaig resultierenden Kostentragungspflichten des Verursachers. So enthält § 25 Abs. 2 Nr. 1 KrWG eine Verordnungsermächtigung zur

31) Abweichend gilt für bereits vor dem 4. 7. 2018 errichteter Regelungsregime bestimmter Produkte eine Verpflichtung schon zum 5. 1. 2023.

32) Siehe neben den einschlägigen Kommentierungen, insbesondere zu § 2 Abs. 2 Nr. 9 KrWG und § 54 WHG, schon aus den 1970er Jahren, kurz nach Inkrafttreten des ersten Abfallgesetzes *Sauter*, ZfW 1974, 213 ff.; ferner BVerwG, Urt. v. 8. 7. 2020 – 7 C 19.18 zur Abgrenzung von Abfall und Abwasser bei der Klärschlammentwässerung.

33) Anhang E Nr. II. 1. Richtlinie 2019/904/EU.

34) Anhang E Nr. III. Richtlinie 2019/904/EU.

35) So treffend *Mann*, in: Versteyl/Mann/Schomerus, KrWG, § 23 Rdnr. 5.

36) Sog. Cradle-to-grave-Prinzip; dazu statt anderer *Kloepfer*, Umweltrecht, § 4 Rdnr. 69 ff.

37) *Tünnesen-Harmes*, in: Jarass/Petersen (Hrsg.), KrWG, § 23 Rdnr. 6.

38) So z. B. Gesetz über das Inverkehrbringen, die Rücknahme und die hochwertige Verwertung von Verpackungen (Verpackungsgesetz – VerpackG) v. 5. 7. 2017, BGBl. I S. 2234; Gesetz über das Inverkehrbringen, die Rücknahme und die umweltverträgliche Entsorgung von Elektro- und Elektronikgeräten (Elektro- und Elektronikgerätegesetz – ElektroG) v. 20. 10. 2015, BGBl. I S. 1739; Gesetz über das Inverkehrbringen, die Rücknahme und die umweltverträgliche Entsorgung von Batterien und Akkumulatoren (Batteriegesetz – BattG) v. 25. 6. 2009, BGBl. I S. 1582; Verordnung über die Überlassung, Rücknahme und umweltverträgliche Entsorgung von Altfahrzeugen (Altfahrzeug-Verordnung – AltfahrzeugV) i. d. F. d. B. v. 21. 6. 2002, BGBl. I S. 2214.

Regelung der Kostenlast für Rücknahme, Verwertung und Beseitigung der zurückzunehmenden Erzeugnisse. Deutlich weiter geht dagegen die noch nicht in deutsches Recht transformierte Kunststoffproduktrichtlinie 2019/904/EU, nach deren Art. 8 Abs. 2 die Mitgliedstaaten dafür Sorge zu tragen haben, dass die Hersteller der tatbestandlich betroffenen Produkte die Kosten tragen, die sich aus den Bestimmungen über die erweiterte Herstellerverantwortung der Richtlinien 2008/98/EG und 94/62/EG[39] ergeben. Darüber hinaus wird die Auferlegung weiterer Kostenlasten vorgeschrieben, unter denen für den hier interessierenden Zusammenhang vor allem auf Art. 8 Abs. 2 lit. b) und c) Richtlinie 2019/904/EU aufmerksam zu machen ist, die Aufwendungen für die Sammlung in öffentlichen Sammelsystemen und für Reinigungsaktionen umfassen. Eine vergleichbare Regelung für weitere Produkte trifft ferner Art. 8 Abs. 3 UAbs. 1 lit. b) der Richtlinie, während in Art. 8 Abs. 4 nähere Vorgaben für die Kostenermittlung aufgestellt werden. Eine diesem Muster nachgebildete Bestimmung für Spurenstoffe im Abwasser wäre jedenfalls rechtssystematisch ohne durchgreifende Bedenken grundsätzlich denkbar.

Bei einer möglichen rechtlichen Umsetzung einer erweiterten Hersteller- und Produktverantwortung im Abwasserrecht sind die Normgeber auf europäischer und nationaler Ebene an allgemeine, übergeordnete Maßstäbe, insbesondere zum Schutz der individuellen Rechtspositionen der Betroffenen gebunden. Die mit einer Anspannung der Pflichtigkeit der Verantwortlichen zwangsläufig einhergehende Einschränkung wirtschaftlicher Gewährleistungen bedingt eine potenziell freiheitsgefährdende Wirkung.[40] Dass sich beispielsweise nach der Entwurfsbegründung der Kommission eine große Mehrheit der Betroffenen für eine Reglementierung ausspricht[41] und dass auch im Rahmen der nationalen Spurenstoffstrategie konsensuale Lösungen zu herstellerbezogenen Maßnahmen angestrebt werden[42], entbindet nicht von den unlängst durch das Bundesverfassungsgericht nachdrücklich in Erinnerung gerufenen verfassungsrechtlichen Anforderungen an grundrechtsrelevante Entscheidungen im Umweltrecht.[43]

### 4. Rechtliche Anforderungen an eine erweiterte Hersteller- und Produktverantwortung im Abwasserrecht

#### *4.1 Wesentliche umweltrechtliche Prinzipien*

##### 4.1.1 Allgemeines

Im originär rechtlichen Sinn wenig ergiebig für die Implementierung einer erweiterten Hersteller- und Produktverantwortung im Abwasserrecht ist zunächst der Rekurs auf die im Kontext der bestehenden erweiterten Herstellerverantwortung im Kreislaufwirtschaftsrecht verbreitet angeführten umweltrechtlichen Prinzipien. Auch wenn diesen mittlerweile überwiegend eine über bloße politische Programmatik hinausgehende rechtliche Verbindlichkeit beigemessen wird[44], bleiben sie in der Allgemeinheit und Schlagworthaftigkeit ihrer normativen Verankerung doch zu unbestimmt, um ihnen präzise Handlungsvorgaben für die umweltrechtliche Legislative und Exekutive zu entnehmen. Insbesondere vermag nicht schon der regelmäßig leicht dargetane Bezug auf sie freiheitsbegrenzende Eingriffe zureichend zu rechtfertigen, wenn es an geeigneten spezifischen gesetzlichen Ermächtigungsgrundlagen fehlt.[45] Im Wesentlichen erschöpft sich ihre rechtliche Relevanz mithin auf die allgemeine Legitimation konkreter normativer Regelungen[46], die ihrerseits den maßgeblichen übergeordneten verfassungs- und unionsrechtlichen Vorgaben zu genügen haben.[47]

Nichts anderes ergibt sich im Übrigen aus der normativen Kodifizierung der Prinzipien im europäischen Primärrecht, das sich – anders als das deutsche Grundgesetz – in Art. 191 Abs. 2 S. 2 AEUV explizit auf Vorsorge und Vorbeugung, auf den Grundsatz, Umweltbeeinträchtigungen mit Vorrang an ihrem Ursprung zu bekämpfen sowie auf das Verursacherprinzip bezieht. Auch diese grundsätzlichen Aussagen im positiven Recht bewegen sich auf einem hohen Abstraktionsniveau[48], das einer unmittelbaren Anwendung entgegensteht.[49] Vielmehr verfügen die zuständigen Rechtssetzungsorgane der Union bei der praktischen Operationalisierung der Prinzipien im sekundären Recht über eine weite Einschätzungsprärogative.[50]

##### 4.1.2 Einzelne Prinzipien

Vor diesem Hintergrund bedürfen die für die Diskussion einer erweiterten Abwasserproduzentenverantwortung einschlägigen umweltrechtlichen Prinzipien an dieser Stelle lediglich der kursorischen Vergewisserung. Strukturell nicht anders als im Kreislaufwirtschaftsrecht verfolgt auch – ungeachtet seiner möglichen konkreten Ausgestaltung *de lege ferenda* – der Ansatz der Implementierung einer Übernahme in das Wasserrecht zur Vermeidung und Verringerung der Einträge von Spurenstoffen in die Gewässer grundsätzlich das Ziel, durch ein Anknüpfen an Herstellungs- oder Verarbeitungsprozesse, die zu einem Eintrag von Spurenstoffen in die Gewässer führen können, schon vor der Entstehung konkreter Gefahren für menschliche Gesundheit und Umwelt diejenigen zu Verhaltens- oder Kostentragungspflichten rechtlich heranzuziehen, denen die entsprechenden Kausalverläufe objektiv zuzurechnen sind. Hiermit werden daher gleich mindestens drei der in Art. 191 Abs. 2 AEUV enthaltenen Grundsätze adressiert: Erstens soll die Lastentragung für die Bekämpfung des Spurenstoffeintrags von der Allgemeinheit auf die hierfür Verantwortlichen übertragen werden und diese so zu gegensteuernden Maßnahmen vor der Einleitung von Abwasser in ein Gewässer oder in eine öffentliche oder private Abwasseranlage oder zur Übernahme der insoweit gebotenen Abwasserbehandlungskosten angehalten werden (Verursacherprinzip). Zweitens wird mit einem tatbestandlichen Bezug auf industrielle oder gewerbliche Her-

39) Richtlinie vom 20.12.1994 über Verpackungen und Verpackungsabfälle, ABl. 1994 L 365, S. 10.
40) So für die Regelungen im KrWG *Kloepfer*, Umweltrecht, § 21 Rdnr. 188; *Konzak*, in: BeckOK Umweltrecht, § 23 KrWG Rdnr. 1; siehe auch zuspitzend *von Lersner*, ZUR Sh 2000, 105: „illiberale Tendenz".
41) COM(2018) final, S. 11.
42) Ergebnispapier Phase 2 des Stakeholder-Dialogs „Spurenstoffstrategie des Bundes", S. 21.
43) BVerfG, Beschl. v. 23.10.2018 – 1 BvR 2523/13, 595/14, BVerfGE 149, 407, 415 f. Rdnr. 24; siehe dazu z. B. *Dolde*, NVwZ 2019, 1567 ff.; *Eichberger*, NVwZ 2019, 1560 ff.; *Reinhardt*, NVwZ 2019, 195 ff.; jeweils m. w. N.
44) Siehe z. B. *Eifert*, Umweltschutzrecht, in: Schoch (Hrsg.), Besonderes Verwaltungsrecht, 2018, Kap. 5 Rdnr. 39 ff.; *Kloepfer*, Umweltrecht, § 4 Rdnr. 17 ff.; *Rehbinder*, in: Rehbinder/Schink (Hrsg.), Grundzüge des Umweltrechts, Kap. 3 Rdnr. 17 f.; *Sparwasser/Engel/Voßkuhle*, Umweltrecht, 5. Aufl. 2003, § 2 Rdnr. 8 ff.
45) *Kloepfer*, Umweltrecht, § 4 Rdnr. 19.
46) Siehe z. B. BVerfG, Beschl. v. 13.3.2007 – 1 BvF 1/05, BVerfGE 118, 79, 110 f.; *Eifert*, in: Schoch (Hrsg.), Besonderes Verwaltungsrecht, Kapitel 5 Rdnr. 40.
47) Dazu unten 4.2.
48) *Nettesheim*, in: Grabitz/Hilf/Nettesheim, Das Recht der Europäischen Union, Loseblattsammlung, Stand: 70. EL 2020, Art. 191 AEUV Rdnr. 83.
49) *Kahl*, in: Streinz (Hrsg.), Vertrag über die Europäische Union, Vertrag über die Arbeitsweise der Europäischen Union, Charta der Grundrechte der Europäischen Union, 3. Aufl. 2018, Art. 191 AEUV Rdnr. 47; ebenso *Calliess*, in: Calliess/Ruffert (Hrsg.), Das Verfassungsrecht der Europäischen Union mit Europäischer Grundrechtecharta, 5. Aufl. 2016, Art. 191 AEUV Rdnr. 51: Erfordernis einer „problembezogenen Optimierung".
50) *Kahl*, in: Streinz (Hrsg.), EUV/AEUV, Art. 191 AEUV Rdnr. 49.

stellungs- und Verarbeitungsprozesse regulativ möglichst nah an der Quelle angeknüpft, um bereits im Stadium der Entstehung einer Gefahr entgegenzuwirken (Ursprungsprinzip, Cradle-to-grave-Prinzip als spezifischer Ausdruck des Vorsorgegedankens). Drittens soll, hiermit verbunden, schon vor Eintritt einer Gefahr oder gar eines Schadens für die menschliche Gesundheit oder die Umwelt rechtlich angesetzt werden, um dem Erfordernis späterer aufwendiger Gegenmaßnahmen oder der Entstehung irreversibler Belastungen effektiv zu begegnen (Vorsorgeprinzip). Im Licht der zentralen umweltrechtlichen Prinzipien korrespondiert daher eine mögliche erweiterte Abwasserproduzentenverantwortung im Wesentlichen mit der bekannten Bewertung der erweiterten Produktverantwortung im geltenden Kreislaufwirtschaftsrecht.[51]

Auf Grund ihres geschilderten grundsätzlichen und abstrakten Charakters und der daraus folgenden Konkretisierungsbedürftigkeit durch spezielles nachgeordnetes Fachrecht auf europäischer oder mitgliedstaatlicher Ebene sind die Prinzipien des europäischen und deutschen Umweltrechts naturgemäß nur sehr eingeschränkt in der Lage, dem zuständigen Normgeber hinreichend klare Voraussetzungen und Grenzen für die Regelung einer erweiterten Hersteller- und Produktverantwortung im Abwasserrecht vorzugeben. So erweist sich insbesondere das in der allgemeinen Diskussion verbreitet als griffig geschätzte Verursacherprinzip bei näherem Hinsehen schon wegen der materiellen Konturenlosigkeit des Verursacherbegriffs selbst als rechtlich kaum steuerungsfähig. So kommen beispielsweise als Verursacher von Spurenstoffen aus Arzneimitteln neben dem Hersteller (als Abwasserproduzent oder durch das Inverkehrbringen des Produkts) auch der Patient (bei bestimmungsgemäßem Gebrauch oder auf Grund unsachgemäßer Entsorgung), der verschreibende oder empfehlende Arzt (auf Grund seiner medizinischen Abwägung zwischen verschiedenen, unterschiedlich gewässerbelastenden Präparaten), der beratende Apotheker (ggfs. auch unter Berücksichtigung eigener unternehmerische Interessen) oder die gesetzlichen oder privaten Krankenversicherungen (auf Grund der Entscheidung über die Erstattungsfähigkeit entsprechender Medikamente) in Betracht.

Tatsächlich maßgebliche materiell-rechtliche Determinanten ergeben sich daher vielmehr aus den jeweils einschlägigen übergeordneten verfassungsrechtlichen Positionen, die im Folgenden am Maßstab des Grundgesetzes nachzuzeichnen sind.

#### *4.2 Materiell-rechtliche Determinanten*

##### 4.2.1 Grundrechte der Hersteller

Die bereits im Rahmen der Vorstellung des kreislaufwirtschaftsrechtlichen Modells erwähnte potenziell freiheitsbeschränkende Wirkung erweiterter Produkt- und Herstellerverantwortlichkeit, die auch schon zu Diskussionen über die Verfassungsmäßigkeit der §§ 23 ff. KrWG geführt haben[52], manifestiert sich in erster Linie in den mit einer solchen Regelung typischerweise verbundenen Eingriffen in Grundrechte der betroffenen Hersteller und Produzenten. Die gesetzliche Anordnung einer erweiterten Verantwortung für Abfall oder auch Abwasser greift dabei vor allem in die Wirtschaftsfreiheit der betroffenen Unternehmen[53], namentlich in die Berufsfreiheit nach Art. 12 GG und die Eigentumsfreiheit nach Art. 14 GG ein.[54] Dabei setzt eine abschließende Bewertung der verfassungsrechtlichen Lage naturgemäß die Prüfung nicht nur der grundsätzlichen gesetzlichen Regelung der erweiterten Verantwortung voraus, sondern erfordert auch die gerade mit Blick auf das Abwasserrecht derzeit naturgemäß nicht zureichend zu antizipierende verfassungsrechtliche Prüfung eines möglichen künftigen konkretisierenden spezifischen Gesetzes- und Verordnungsrechts. Vor diesem Hintergrund müssen sich die nachfolgenden Ausführungen zwangsläufig auf die Erörterung der maßgeblichen Eckwerte beschränken und stehen unter dem Vorbehalt einer späteren Verifizierung auf der Grundlage zureichend fassbarer Regelungskonzepte und Rechtssetzungsentwürfe eines entsprechenden abwasserrechtlichen Konstrukts.

Für den Regelfall dürfte mit Blick auf die von einer künftigen erweiterten Abwasserproduzentenverantwortung berührten Grundrechte betroffener Unternehmen davon auszugehen sein, dass sich bereichsspezifische Konkretisierungen in berufsrechtlicher Sicht als Berufsausübungsregeln[55] und in eigentumsrechtlicher Sicht als grundsätzlich entschädigungslos hinzunehmende Inhalts- und Schrankenbestimmungen[56] darstellen, so dass jeweils einfache Verhältnismäßigkeitsprüfungen unter Berücksichtigung der einschlägigen grundrechtsspezifischen Ausprägungen durchzuführen sind.[57] Erst bei Entzug konkreter Eigentumspositionen ohne Güterbeschaffungszweck sind nach der Rechtsprechung des Bundesverfassungsgerichts gesteigerte Verhältnismäßigkeitsmaßstäbe anzulegen und gegebenenfalls auch Entschädigungsansprüche der Betroffenen zu erwägen.[58] Ein grundsätzlich überwiegendes Vertrauen der Unternehmen, von einer sich dynamisch fortentwickelnden Anspannung abwasserrechtlicher Pflichten verschont zu bleiben, besteht dabei grundsätzlich nicht[59], doch sind Einschränkungen eines bereits bestehenden Betriebs durch Einführung oder Intensivierung der erweiterten Herstellerverantwortung auch an den verfassungsrechtlichen Vorgaben des rechtsstaatlichen Rückwirkungsverbots[60] zu messen.[61]

##### 4.2.2 Grundrechtsschranken

Im Rahmen der angesprochenen Verhältnismäßigkeitsprüfungen sind die mit der Einführung einer erweiterten Hersteller- und Produktverantwortung im Abwasserrecht verbundenen Grundrechtseingriffe in Berufs- und Eigentumsfreiheit verfassungsrechtlich gerechtfertigt, wenn und soweit die so geschützten Rechtspositionen in der Abwägung mit maßgeblichen gegenläufigen Belangen zurück-

51) Siehe dazu z. B. *Kloepfer*, Umweltrecht, § 21 Rdnr. 187; *Konzak*, in: BeckOK, Umweltrecht, § 23 KrWG Rdnr. 4; *Tünnesen-Harmes*, in: Jarass/Petersen (Hrsg.), KrWG, § 23 Rdnr. 1; siehe auch *Mann*, in: Versteyl/Mann/Schomerus, KrWG, § 23 Rdnr. 1 zum Prinzip der Ressourcenschonung.

52) *von Lersner*, ZUR Sh 2000, 105, 109; *Prelle*, in: Schmehl/Klement (Hrsg.), KrWG, § 23 Rdnr. 9 ff.

53) *Kloepfer*, Umweltrecht, § 21 Rdnr. 188; *Konzak*, in: BeckOK Umweltrecht, § 23 KrWG Rdnr. 1.

54) *Prelle*, in: Schmehl/Klement (Hrsg.), KrWG, § 23 Rdnr. 9.

55) Siehe dazu statt anderer *Manssen*, in: von Mangoldt/Klein/Starck, Grundgesetz, Kommentar, Bd. 1, 7. Aufl. 2018, Art. 12 Rdnr. 66 ff., 157 ff.; *Wieland*, in: Dreier (Hrsg.) Grundgesetz, Kommentar, Bd. I, 3. Aufl. 2013, Art. 12 Rdnr. 69 ff. jeweils m. w. N.

56) Art. 14 Abs. 1 S. 2 GG; siehe dazu statt anderer *Depenheuer/Froese*, in: von Mangoldt/Klein/Starck, GG, Art. 14 Rdnr. 222 ff.; *Wieland*, in: Dreier (Hrsg.), GG, Art. 14 Rdnr. 90 ff., 103 ff. jeweils m. w. N.

57) Siehe zur parallelen Bewertung im Kreislaufwirtschaftsrecht *von Lersner*, ZUR Sh 2000, 105, 109 f.; *Mann*, in: Versteyl/Mann/Schomerus, KrWG, § 23 Rdnr. 8; *Prelle*, in: Schmehl/Klement (Hrsg.), KrWG, § 23 Rdnr. 11; *Tünnesen-Harmes*, in: Jarass/Petersen (Hrsg.), KrWG, § 23 Rdnr. 7.

58) BVerfG, Urt. v. 6. 12. 2016 – 1 BvR 2821/11, 321, 1456/12, BVerfGE 143, 246, 341 ff. Rdnr. 268 ff. m. w. N.

59) *Mann*, in: Versteyl/Mann/Schomerus, KrWG, § 23 Rdnr. 8; *Tünnesen-Harmes*, in: Jarass/Petersen (Hrsg.), KrWG, § 23 Rdnr. 7.

60) Siehe dazu z. B. *Schulze-Fielitz*, in: Dreier (Hrsg.), Grundgesetz, Kommentar, Bd. II, 3. Aufl. 2015, Art. 20 (Rechtsstaat) Rdnr. 151 ff.; *Sommermann*, in: von Mangoldt/Klein/Starck, Grundgesetz, Kommentar, Bd. 2, 7. Aufl. 2018, Art. 20 Rdnr. 292 ff. jeweils m. w. N.

61) So zum Kreislaufwirtschaftsrecht *Prelle*, in: Schmehl/Klement (Hrsg.), KrWG, § 23 Rdnr. 10.

treten. Vorrangig zu nennen sind insoweit die jeweils mit Verfassungsrang ausgestatteten Ziele der rechtlichen Bekämpfung des Eintrags von Spurenstoffen in die Gewässer im Interesse des Schutzes der menschlichen Gesundheit vor Beeinträchtigungen infolge der Aufnahme der Stoffe, insbesondere über das Trinkwasser, und der Gewässer als Bestandteil des Naturhaushalts und als Lebensraum für Tiere und Pflanzen.[62] Gemäß Art. 2 Abs. 2 S. 1 GG hat jeder das Recht auf Leben und körperliche Unversehrtheit, wovon auch die körperliche Gesundheit im engeren biologisch-physiologischen Sinn erfasst wird.[63] Das Grundrecht wirkt hierbei nicht allein als konstitutionelle Abwehr gegenüber staatlichen Eingriffen, sondern verpflichtet den Staat zugleich, sich aktiv schützend und fördernd vor das Rechtsgut zu stellen, um es vor Beeinträchtigungen durch Dritte zu bewahren (staatliche Schutzpflicht).[64] Art. 20a GG enthält sodann – wenngleich in höchst abstrakter Ausgestaltung – den Verfassungsauftrag des Schutzes der Umwelt als natürliche Lebensgrundlage des Menschen und der Tiere.

Insofern fällt eine Rechtfertigung des Eingriffs in die Wirtschaftsfreiheiten des Grundgesetzes vergleichsweise leicht, soweit die in den Blick genommenen Stoffe eine nachweisliche Gefahr für die menschliche Gesundheit oder die Umwelt begründen. Umgekehrt trägt die Auferlegung einer erweiterten Abwasserproduzentenverantwortung zur Eliminierung von Spurenstoffen im Gewässer verfassungsrechtlich nicht, wenn und soweit von diesen weder eine Gefahr für die menschliche Gesundheit noch für die Umwelt ausgehen. Entsprechende Eingriffe wären insoweit zur Zielerreichung nicht geeignet und erforderlich und damit verfassungsrechtlich nicht gerechtfertigt.

Derzeit ist indes die Beurteilung der von Spurenstoffen ausgehenden Wirkungen auf die genannten Schutzgüter vielfach (noch) nicht naturwissenschaftlich verlässlich möglich, sondern ist vielmehr durch ein hohes Maß an tatsächlicher Unsicherheit gekennzeichnet, da für zahlreiche Stoffe eine unzureichende Studienlage zu konstatieren ist.[65] Daraus folgt nach dem zuvor Gesagten zum einen eine rechtlich nicht ohne weiteres tragfähige Rechtfertigung für den Eingriff in grundrechtlich geschützte Positionen, zum anderen aber auch die Erwartung an eine dynamische normative Regelung, mit der Entwicklung der naturwissenschaftlichen Erkenntnis angemessen Schritt halten zu können.

Allerdings ist der Normgeber gerade im Umwelt- und Technikrecht nicht darauf beschränkt, erst tätig zu werden, wenn eine nachweisliche, konkrete Gefahr für ein bestimmtes Schutzgut besteht, sondern kann nach Vornahme einer Gesamtabwägung vorsorglich auch schon dann grundrechtsbeschränkende Regelungen treffen, wenn dies im Verhältnis von Schwere des Grundrechtseingriffs und Bedeutung des verfolgten öffentlichen Interesses gerechtfertigt ist.[66] Erst Vorverlagerungen der Reglementierungen bei Ungewissheiten jenseits einer Schwelle praktischer Vernunft sind als unentrinnbares Restrisiko als sozialadäquate Lasten vom Bürger zu tragen.[67] Ein Streben nach absoluter Sicherheit durch Elimination jeglicher, mit den modernen naturwissenschaftlichen Methoden nachweisbarer, nicht natürlicher Einträge von Spurenstoffen in die Gewässer ist verfassungsrechtlich nicht gerechtfertigt.[68]

Die sohin bei möglicher Einführung einer erweiterten Abwasserproduzentenverantwortung gebotene verhältnismäßigkeitsrechtliche Abwägung wird dabei zuvörderst auf die Bewertung der objektiven Gefahren- und Risikopotenziale der verschiedenen Spurenstoffe zu stützen sein. Darüber hinaus werden aber auch weitere Belange wie beispielsweise Zustand und Vorbelastung der örtlich betroffenen Vorfluter sowie Bestand und Perspektiven regionaler Infra- und Wirtschaftsstrukturen in die Entscheidung einzubeziehen sein. Zudem kann gerade bei den spurenstoffimmanenten Belastungen in geringem quantitativen Umfang die Identifikation und Bewertung unterschiedlicher Risikogruppen wie u.a. werdende Mütter, Kinder oder allergiekranke Menschen geboten sein.[69] Nicht zuletzt ist auch die wirtschaftliche Zumutbarkeit der mit der erweiterten Abwasserproduzentenverantwortung verbundenen Handlungs- oder Zahlungspflichten der betroffenen Grundrechtsträger zwingend in die Verhältnismäßigkeitsprüfung einzubeziehen.[70] Eine Regelung auf bloßen Verdacht ist dagegen verfassungsrechtlich nicht möglich.

#### *4.3 Normative Umsetzung*

In normsetzungstechnischer Hinsicht ist an dieser Stelle auch kurz auf die Optionen einer rechtlichen Umsetzung einer erweiterten Hersteller- und Produktverantwortung im Abwasserrecht einzugehen. Grundsätzlich erscheint dabei sowohl eine Implementierung im Recht der Europäischen Union als auch im deutschen Recht denkbar. Auf europäischer Ebene wäre eine entsprechende Regelung als Maßnahme des Umwelt- und Gesundheitsschutzes vom Kompetenztitel des Art. 191 AEUV getragen. Ergänzt werden könnte eine eigenständige richtlinienrechtliche Bestimmung, die in Anlehnung an die erwähnten Artikel im europäischen Abfallrecht[71] den Mitgliedstaaten bestimmte Ziele für die Formulierung von Herstellerpflichten im Abwasserrecht vorschreibt. Rein formaler Natur ist sodann die systematische Zuordnung im Kontext des geltenden europäischen Wasserrechts, die ohne rechtlich erhebliche inhaltliche Unterschiede ebenso gut in der allgemeineren Kodifizierung der Wasserrahmenrichtlinie[72] wie auch in der spezielleren kommunalen Abwasserrichtlinie[73] erfolgen könnte. Zudem befinden sich beide Richtlinien derzeit im Stadium der Evaluation durch die Europäische Kommission mit der möglichen Folge einer anschließenden Novellierung, so dass sich eine geeignete Einspeisung der konzeptionellen Vorstellungen in den noch nicht ab-

62) Siehe auch die Zielsetzungen des § 1 WHG.
63) BVerfG, Beschl. v. 14. 1. 1981 – 1 BvR 612/72, BVerfGE 56, 54, 74 f. unter Hinweis auf das Begriffsverständnis der WHO; ferner *Schulze-Fielitz*, in: Dreier (Hrsg.), GG, Art. 2 II Rdnr. 33 ff.; *Starck*, in: von Mangoldt/Klein/Starck, GG, Art. 2 Rdnr. 193.
64) BVerfG, Urt. v. 25. 2. 1975 – 1 BvF 1, 2, 3, 4, 5, 6/74, BVerfGE 39, 1, 41 ff.; BVerfG, Urt. v. 16. 10. 1977 – 1 BvQ 5/77, BVerfGE 46, 160, 164; BVerfG, Beschl. v. 8. 8. 1978, 2 BvL 8/77, BVerfGE 49, 89, 140 ff.
65) Ergebnispapier Phase 2 des Stakeholder-Dialogs „Spurenstoffstrategie des Bundes", S. 1; ferner *Gawel/Schindler*, ZUR 2015, 387; *Kern*, ZUR 2011, 9 f.; *Peine*, NuR 2015, 822; *Reinhardt*, LKV 2018, 289, 297.
66) Siehe dazu allgemein *Dreier*, in: Dreier (Hrsg.), GG, Vorb. Rdnr. 149; *Herdegen*, in: Maunz/Dürig, Grundgesetz, Kommentar, Loseblattsammlung, Stand: 87. EL 2019, Art. 1 Abs. 3 Rdnr. 47; aus spezifisch umweltrechtlicher Sicht *Salzwedel*, in: Isensee/Kirchhof (Hrsg.), HStR IV, § 97 Rdnr. 15 ff. jeweils m. w. N.
67) So BVerfG, Beschl. v. 8. 8. 1978, 2 BvL 8/77, BVerfGE 49, 89, 90 zum Vorsorgeniveau im Atomrecht.
68) Allgemein BVerfG, Beschl. v. 8. 8. 1978, 2 BvL 8/77, BVerfGE 49, 89, 143; BVerfG, Beschl. v. 18. 2. 2010 – 2 BvR 2502/08, NVwZ 2010, S. 702, 704; *Salzwedel*, in: Isensee/Kirchhof (Hrsg.), HStR IV, § 97 Rdnr. 12.
69) Dazu *Salzwedel*, in: Isensee/Kirchhof (Hrsg.), HStR IV, § 97 Rdnr. 12 zum allgemeinen Lebensrisiko.
70) So zur erweiterten Produktverantwortung im Kreislaufwirtschaftsrecht *von Lersner*, ZUR Sh 2000, 105, 109; allgemein *Heselhaus*, Verfassungsrechtliche Grundlagen des Umweltschutzes, in: Rehbinder/Schink (Hrsg.), Grundzüge des Umweltrechts, Kapitel 1 Rdnr. 116 ff.; *Kloepfer*, Umweltrecht, § 3 Rdnr. 91 ff.; *Schulze-Fielitz*, in: Dreier (Hrsg.), GG, Art. 20 (Rechtsstaat) Rdnr. 184.
71) Dazu oben 3.3.
72) Richtlinie 2000/60/EG vom 23. 10. 2000 zur Schaffung eines Ordnungsrahmens für Maßnahmen der Gemeinschaft im Bereich der Wasserpolitik, ABl. 2000 L 327, S. 1.
73) Richtlinie 91/271/EWG vom 21. 5. 1991 über die Behandlung von kommunalem Abwasser, ABl. 1991 L 135, S. 40.

geschlossenen Rechtssetzungsprozess auch praktisch gut denken ließe. Möglicherweise legen freilich die politisch durchaus kontroverseren Diskussionen um die Reform der Wasserrahmenrichtlinie eine Konzentration auf die anstehende Fortschreibung der kommunalen Abwasserrichtlinie eher nahe. In zeitlicher Hinsicht ist bei einer europäischen Lösung im Übrigen auch an die Transformationsfrist für den nationalen Gesetz- und Verordnungsgeber zu erinnern, die die Geltung des neuen Rechts zeitlich weiter verzögern würde.

Im deutschen Verfassungsrecht steht dem Bund gemäß Art. 74 Abs. 1 Nr. 32 GG die konkurrierende Gesetzgebungskompetenz für den Wasserhaushalt zu, die ohne weiteres auch die Ergänzung einer erweiterten Abwasserproduzentenverantwortung in den §§ 54 ff. WHG einschließlich der Schaffung zugehöriger Verordnungsermächtigungen für bereichsspezifische Anforderungen etwa in den Bereichen Arzneimittel, Kunststoffe, Biozide etc. umfasst. Als mindestens stoffbezogene Regelung im Sinne des Art. 72 Abs. 3 Nr. 5 WHG unterläge ein derartiges Bundesrecht zudem nicht der verfassungsrechtlichen Abweichungsbefugnis[74] der Länder. Die mit einer solchen Verpflichtung der verantwortlichen Unternehmen verbundene materielle Anspannung nationaler Gewässerschutzstandards ist schließlich unter den Voraussetzungen des Art. 193 S. 1, 2 AEUV auch unionsrechtlich grundsätzlich unbedenklich, soweit dies mit den Verträgen vereinbar ist, d. h. soweit nicht durch einzelne Regelungen die Erfordernisse des europäischen Binnenmarkts, das Diskriminierungsverbot oder andere primärrechtliche Positionen[75] verletzt werden. Da indes zu erwarten ist, dass im Fall einer rein nationalen Lösung die Geltungsbereich des Wasserhaushaltsgesetzes tätigen Unternehmen einen standortbezogenen Wettbewerbsnachteil geltend machen dürften, erscheint eine europäische Lösung im Ergebnis vorzugswürdig.[76] Damit könnten zudem mögliche Konflikte mit der primärrechtlichen Warenverkehrsfreiheit nach den Art. 34 ff. AEUV, etwa auf Grund rein nationaler Anforderungen an Produktionsverfahren und Produktdesign vermieden werden.

#### *4.4 Bewertung: Verhältnismäßigkeit im europäischen Kontext*

Naturgemäß fällt eine abschließende resümierende Bewertung einer möglichen künftigen erweiterten Hersteller- und Produktverantwortung in diesem vergleichsweise frühen Stadium schwer. Wenig ergiebig erweist sich dabei zunächst der gefällige und verbreitete Rekurs auf die mit der Anspannung der individuellen Verantwortlichkeit adressierten umweltrechtlichen Prinzipien. Verursacher-, Ursprungs- und Vorsorgeprinzip sind thematisch einschlägig, sind aber auf Grund ihres Rechtscharakters und ihres hohen Abstraktionsgrads nicht in der Lage, die Einführung einer derartigen Regelung zu erzwingen, abschließend zu rechtfertigen oder inhaltlich hinreichend präzise zu steuern. Vielmehr sind sie auf die Operationalisierung durch spezifische gesetzliche oder verordnungsrechtliche Regelungen angewiesen, die ihrerseits den übergeordneten Vorgaben, insbesondere den einschlägigen verfassungsrechtlichen Gewährleistungen und Zielsetzungen zu genügen haben. Im Vordergrund stehen dabei die wirtschaftlichen Freiheiten der von einer angespannten Verantwortlichkeit betroffenen Unternehmen, die sich nach deutschem Verfassungsrecht insbesondere aus den Grundrechten der Art. 12, 14 GG ergeben. Eingriffe in Berufs- und Eigentumsfreiheit können dabei indes grundsätzlich mit den ebenfalls über Verfassungsrang verfügenden Positionen der menschlichen Gesundheit nach Art. 2 Abs. 2 GG und des Umweltschutzes nach Art. 20a GG gerechtfertigt werden. Das Beispiel der Produktverantwortung im Kreislaufwirtschaftsrecht lehrt insoweit, dass die einzelnen Bestandteile der rechtlichen Verpflichtung jeweils einem Verhältnismäßigkeitstest standzuhalten haben, in dem die einander widerstreitenden Belange gegeneinander abzuwägen sind. Dabei werden Extrempositionen wie die vollständige Eliminierung jeglicher tatsächlich messbarer Spurenstoffe ungeachtet ihrer möglichen nachteiligen Auswirkungen auf menschliche Gesundheit und Umwelt oder ein Streben nach absoluter Sicherheit von vornherein auszuschließen sein. Im Übrigen bedarf es der differenzierten Bewertung und normativen Umsetzung. Dabei wird etwa zum einen eine nach Stoffen und Stoffgruppen (z. B. Rückstände von Kunststoffprodukten, Arzneimitteln, Pflanzenschutzmitteln, Bioziden) unterscheidende Ausführungsgesetzgebung erforderlich, zum anderen sind die in Erwägung gezogenen Maßnahmen wie die Verpflichtung zur Entwicklung spurenstoffloser oder -armer Verfahrensweisen, zur betrieblichen Vorklärung oder zur finanziellen Beteiligung am kommunalen Aufwand der Abwasserbehandlung in ihrer jeweils beabsichtigten Ausformung auf Geeignetheit, Erforderlichkeit und Angemessenheit hin zu überprüfen. In verbandskompetenzrechtlicher Hinsicht erscheint eine Implementierung sowohl auf unionsrechtlicher als auch auf deutscher Regelungsebene denkbar, wobei sich aus praktischen und rechtlichen Gründen eine entsprechende Erweiterung der kommunalen Abwasserrichtlinie am ehesten anbietet.

### 5. Zusammenfassung

(1) Das gegenwärtig intensiv diskutierte Problem des Eintrags von Spurenstoffen in die Gewässer ist im geltenden Wasserrecht bislang nicht zureichend bewältigt. Da ausschließlich ordnungsrechtliche Konzepte aus tatsächlichen und rechtssetzungstechnischen Gründen nur eingeschränkte Abhilfe versprechen und auch ein auf den gesetzlichen Bewirtschaftungsauftrag der staatlichen Wasserwirtschaftsverwaltung gegründeter Ansatz nicht zu tragen vermag, werden derzeit unterschiedliche alternative Modelle der rechtlichen Verhaltenssteuerung erwogen.

(2) Unter diesen kann das Konzept einer erweiterten individuellen Inpflichtnahme der für den Spurenstoffeintrag Verantwortlichen auf ein musterhaftes Konstrukt im europäischen und deutschen Kreislaufwirtschafts- und Abfallrecht aufbauen. Die fließenden Grenzen zwischen den Rechtsbegriffen Abfall und Abwasser, die auf eine letztlich artifizielle normative Abgrenzung verschiedener Rechtsregimes zurückzuführen sind, legen eine grundsätzliche Orientierung an den kreislaufwirtschaftsrechtlichen Regelungen auch für das Abwasserrecht durchaus nahe.

(3) In inhaltlicher Hinsicht ist zunächst der verbreitete Rekurs auf einschlägige Prinzipien des Umweltrechts zur Rechtfertigung oder gar inhaltlichen Determinierung des Konzepts einer erweiterten Hersteller- und Produktverantwortung im Abwasserrecht wenig hilfreich. Auch wenn hierdurch durchaus gleich mehrere Prinzipien thematisch berührt werden, bleiben diese doch trotz ihrer grundsätzlichen Rechtsverbindlichkeit im Ergebnis steuerungstheoretisch viel zu abstrakt und bedürfen vielmehr erst der bereichsspezifischen Operationalisierung durch konkretisierendes Gesetzes- und Verordnungsrecht.

(4) Bei der gesetzlichen Ausgestaltung einer erweiterten Hersteller- und Produktverantwortung im Abwasser-

74) Dazu neben den Kommentierungen des Art. 72 GG *Reinhardt*, AöR 135, 2020, 459, 482 ff.

75) Näher *Calliess*, in: Calliess/Ruffert (Hrsg.), EUV/AEUV, Art. 193 AEUV Rdnr. 11 ff. m. w. N.

76) Dem entspricht die in der Begründung des Kommissionsentwurfs zur Kunststoffproduktrichtlinie 2019/904/EU mitgeteilte überwiegende Ansicht der konsultierten Interessenträger, nach der Maßnahmen, die nicht auf EU-Ebene ergriffen werden, wirkungslos wären, COM(2018) final, S. 11.

recht durch Begründung besonderer Verhaltens- und Kostentragungspflichten *de lege ferenda* sind insbesondere die damit notwendig verbundenen Eingriffe in grundrechtsgeschützte Positionen der betroffenen Unternehmen zu bedenken. Diese können grundsätzlich durch gegenläufige Belange des Schutzes der menschlichen Gesundheit und der Umwelt gerechtfertigt werden. Damit werden komplexe Verhältnismäßigkeitsprüfungen in Rechtssetzung und Rechtsanwendung erforderlich, die nicht pauschal antizipiert werden können, sondern unter Berücksichtigung des konkret nachweislichen oder belastbar prognostizierten Gefahren- und Risikopotenzials der verschiedenen Stoffe und Stoffgruppen für die konkret in Erwägung gezogenen einzelnen erweiterten Rechtspflichten der betroffenen Hersteller vorzunehmen sind.

(5) Eine erweiterte Hersteller- und Produktverantwortung im Abwasserrecht ist kompetenzrechtlich sowohl auf unionsrechtlicher als auch auf bundesrechtlicher Ebene möglich. Insbesondere mit Rücksicht auf den grenzüberschreitenden Wettbewerb erscheint dabei eine europäische Lösung vorzugswürdig. Dabei würde sich in rechtssetzungstechnischer Hinsicht eine entsprechende Regelung nach dem Vorbild des europäischen Abfallrechts als Ergänzung der kommunalen Abwasserrichtlinie anbieten.

**Open Access.** Dieser Artikel wird unter der Creative Commons Namensnennung 4.0 International Lizenz veröffentlicht, welche die Nutzung, Vervielfältigung, Bearbeitung, Verbreitung und Wiedergabe in jeglichem Medium und Format erlaubt, sofern Sie den/die ursprünglichen Autor(en) und die Quelle ordnungsgemäß nennen, einen Link zur Creative Commons Lizenz beifügen und angeben, ob Änderungen vorgenommen wurden.
Die in diesem Artikel enthaltenen Bilder und sonstiges Drittmaterial unterliegen ebenfalls der genannten Creative Commons Lizenz, sofern sich aus der Abbildungslegende nichts anderes ergibt. Sofern das betreffende Material nicht unter der genannten Creative Commons Lizenz steht und die betreffende Handlung nicht nach gesetzlichen Vorschriften erlaubt ist, ist für die oben aufgeführten Weiterverwendungen des Materials die Einwilligung des jeweiligen Rechteinhabers einzuholen.
Weitere Details zur Lizenz entnehmen Sie bitte der Lizenzinformation auf http://creativecommons.org/licenses/by/4.0/deed.de.

Open Access funding enabled and organized by Projekt DEAL.

## Das Vorsorgeprinzip beim Ausbau von G5

**Hans-Jürgen Müggenborg**

Der Ausbau des neuen Mobilfunkstandards 5G steht in den Startlöchern und hat mancherorts bereits begonnen. Zu fragen ist, ob der weitgehend ungeregelte Ausbau dieser neuen Technologie vor dem Hintergrund zahlreicher Untersuchungen, die auf ein gesundheitliches Gefährdungspotenzial der Technik hinweisen, überhaupt zulässig ist.

### 1. Problemaufriss

Hohe Übertragungsraten und schnelles Internet haben unbestreitbar Vorteile. Deshalb sehen manche Branchen dem zügigen Ausbau des neuen Mobilfunknetzes 5G, die auch als Schlüsseltechnologie für den digitalen Wandel bezeichnet wird, mit hohen Erwartungen entgegen. Autonomes Fahren, eHealth[1] oder das Internet-of-Things etwa würden ohne 5G nicht funktionieren.

Auf der anderen Seite mehren sich Stimmen, die vor den negativen Folgen von 5G warnen.[2] Befürchtet werden gesundheitliche Schäden durch die Funkstrahlung, angefangen von Schlafstörungen bis hin zu Krebs. Auf der anderen Seite wird darauf hingewiesen, dass es weltweit keine Studie gibt, die die Unschädlichkeit der 5G-Mikrowellen-Funktechnologie belegt, wohl aber zahlreiche Studien, die vor den gesundheitlichen Folgen warnen. Namhafte Ärzte haben in einem offenen Brief vom 6. 4. 2020 an Bundeskanzlerin Angela Merkel auf eine Schwächung des Immunsystems durch 5G-Strahlung hingewiesen.[3] Es gibt ferner den vom IFUR – Privates Institut für Urfeldforschung von Wolfgang Kühl am 24. 4. 2020 veröffentlichten Verdacht, dass die hohen 60 GHZ-Frequenzen des 5G-Bandes zu einer physikalischen Veränderung von Sauerstoffmolekülen führen, so dass der Sauerstoff vom Blut nicht mehr problemlos transportiert werden kann.[4] Zudem wird der Energiehunger der Welt bei einem flächendeckenden 5G-Ausbau rapide steigen. Der dazu nötige Strombedarf wird, solange die Energie nicht zu 100 % aus erneuerbaren Energien gewonnen wird[5], nicht ohne weitere Belastungen für die Umwelt gedeckt werden können.

Prof. Dr. jur. Hans-Jürgen Müggenborg,
Rechtsanwalt und Fachanwalt für Verwaltungsrecht,
Aachen, Deutschland

1) Siehe dazu das eHealth-Gesetz: Gesetz für sichere digitale Kommunikation und Anwendungen im Gesundheitswesen sowie Änderung weiterer Gesetze v. 21. 12. 2015, BGBl. I S. 2408.

2) *Budzinski*, NuR 2008, 535 ff. *ders.*, NVwZ 2010, 1205 ff.; *ders.*, NVwZ 2012, 547 ff.; *ders.*, NVwZ 2012, 1593 ff.; *ders.*, NVwZ 2013, 404 ff.; *ders.*, NuR 2013, 613 ff.; *ders.*, NVwZ 2013, 988 ff.; *ders.*, NuR 2017, 757 ff.; *ders.*, WuW 2019, 508 ff.; *Budzinski/Hutter*, NVwZ 2014, 418 ff.; *Budzinski/Kühling*, NVwZ 2015, 1410 ff.; *Wahlfels*, NVwZ 2013, 653 ff.; *Appel/Bulla*, DVBl. 2008, 1277 ff.; *Gratzel/Kniep*, WuM 2009, 383 ff.; *Kniep*, ZMR 2009, 510 ff.; *Gehrken/Kahle/Mechel*, ZUR 2006, 72 ff.; *Koch*, NVwZ 2013, 251 ff.

3) Offener Brief an die Bundesregierung, Stand 17. 11. 2020, abrufbar unter https://www.weisse-zone-rhoen.de/5g/offener-brief-an-die-bundesregierung/.

4) Weitere Informationen, Stand 17. 11. 2020, abrufbar unter https://ifurinstitut.wordpress.com/2020/04/24/corona-covid-19-symptoms-caused-by-60-ghz-frequencies/.

5) Nach einer Auswertung des Umweltbundesamtes liegt der Anteil der erneuerbaren Energien am Gesamtenergieverbrauch der Bundesrepublik im Jahr 2019 bei 17,1 %; betrachtet man nur die Stromproduktion liegt der Anteil erneuerbarer Energien im Jahr 2019 bei 42,1 %; Umweltbundesamt, Erneuerbare Energien in Deutschland – Daten zur Entwicklung im Jahr 2019, Stand 17. 11. 2020, abrufbar unter https://www.umweltbundesamt.de/sites/default/files/medien/1410/publikationen/2020-04-03_hgp-ee-in-zahlen_bf.pdf.
